# Disynaptic Inhibitory Cerebellar Control Over Caudal Medial Accessory Olive

**DOI:** 10.1523/ENEURO.0262-23.2023

**Published:** 2024-02-09

**Authors:** Willem S. van Hoogstraten, Marit C. C. Lute, Zhiqiang Liu, Robin Broersen, Luca Mangili, Lieke Kros, Zhenyu Gao, Xiaolu Wang, Arn M. J. M. van den Maagdenberg, Chris I. De Zeeuw

**Affiliations:** ^1^Department of Neuroscience, Erasmus MC, Rotterdam 3015 GD, The Netherlands; ^2^Netherlands Institute for Neuroscience, Amsterdam 1105 BA, The Netherlands; ^3^Departments of Neurology, Leiden University Medical Center, Leiden 2333 ZA, The Netherlands; ^4^Human Genetics, Leiden University Medical Center, Leiden 2333 ZA, The Netherlands

**Keywords:** cerebellum, gaze control, inferior olive, Medial accessory olive, olivocerebellar system, superior colliculus

## Abstract

The olivocerebellar system, which is critical for sensorimotor performance and learning, functions through modules with feedback loops. The main feedback to the inferior olive comes from the cerebellar nuclei (CN), which are predominantly GABAergic and contralateral. However, for the subnucleus d of the caudomedial accessory olive (cdMAO), a crucial region for oculomotor and upper body movements, the source of GABAergic input has yet to be identified. Here, we demonstrate the existence of a disynaptic inhibitory projection from the medial CN (MCN) to the cdMAO via the superior colliculus (SC) by exploiting retrograde, anterograde, and transsynaptic viral tracing at the light microscopic level as well as anterograde classical and viral tracing combined with immunocytochemistry at the electron microscopic level. Retrograde tracing in Gad2-Cre mice reveals that the cdMAO receives GABAergic input from the contralateral SC. Anterograde transsynaptic tracing uncovered that the SC neurons receiving input from the contralateral MCN provide predominantly inhibitory projections to contralateral cdMAO, ipsilateral to the MCN. Following ultrastructural analysis of the monosynaptic projection about half of the SC terminals within the contralateral cdMAO are GABAergic. The disynaptic GABAergic projection from the MCN to the ipsilateral cdMAO mirrors that of the monosynaptic excitatory projection from the MCN to the contralateral cdMAO. Thus, while completing the map of inhibitory inputs to the olivary subnuclei, we established that the MCN inhibits the cdMAO via the contralateral SC, highlighting a potential push–pull mechanism in directional gaze control that appears unique in terms of laterality and polarity among olivocerebellar modules.

## Significance Statement

All dendritic spines of the inferior olive receive a combined inhibitory and excitatory input, which is critical for regulation of temporal control during sensorimotor coordination. Yet, some sources of GABAergic input to specific parts of the olive remain to be identified. While completing the map of inhibitory inputs, we establish here that part of the caudal medial accessory olive receives a GABAergic input from neurons in the contralateral superior colliculus that receive a direct input from the cerebellar nuclei. Interestingly, the laterality of this disynaptic projection is unique among olivocerebellar modules, highlighting a potential push–pull mechanism in directional gaze control.

## Introduction

The olivocerebellar system plays a critical role in sensorimotor coordination (De Zeeuw et al., 2011; Llinas, 2013). Connections between Purkinje cells in cerebellar cortex, neurons in cerebellar nuclei (CN) and neurons in inferior olive (IO) are organized in olivocerebellar modules, with each module controlling one or more specific behaviors (Apps et al., 2018; De Zeeuw, 2021). The main feedback pathway concerns the projection from CN to IO, which is predominantly GABAergic and contralateral (De Zeeuw et al., 1988, 1993, 1994a; Fredette and Mugnaini, 1991). However, a recent study has uncovered that the projection from the medial cerebellar nucleus (MCN) to the contralateral caudal Medial Accessory Olive (cMAO) is predominantly excitatory rather than inhibitory (Wang et al., 2023), which is an exception to the longstanding view that the CN can only inhibit the IO. More specifically, in line with the recent dissection of the connections within the olivocerebellar system (Fujita et al., 2020), this excitatory projection ends up in the subnucleus d of the cMAO (cdMAO). The reason for this exception to the norm of inhibitory CN-IO projections could be that the cdMAO is involved in control of directional saccade and upper body movements (Wang et al., 2023), which may require feedback signals of opposite polarity. Given that all dendrites of all olivary neurons are studded with GABAergic terminals (De Zeeuw et al., 1989, 1990a,b; Ruigrok et al., 1990) and that all olivary subnuclei receive a GABAergic input (Nelson et al., 1989), the finding of the excitatory MCN projection to the cdMAO raises the question what the source is of GABAergic input to the cdMAO. The combined inhibitory and excitatory input to dendritic spines in the IO facilitates its neurons to fine-tune the timing of its climbing fiber activity (Loyola et al., 2023), allowing for precise control of online motor performance (Welsh et al., 1995; Van Der Giessen et al., 2008; Hoogland et al., 2015) and long-term adjustments (Gao et al., 2012; Kostadinov et al., 2019; Bina et al., 2022).

We therefore set out to uncover the origin of the GABAergic input to the cdMAO, working toward completing the topographic map of GABAergic inputs to the olivary subnuclei (Nelson et al., 1989). None of the typical sources of inhibition to the IO in the hindbrain have been found to provide a GABAergic input to the cdMAO (Barmack et al., 1993; De Zeeuw et al., 1993, 1994a), requiring us to evaluate a broader range of brain areas. A possible answer lies in more rostral areas in the midbrain that have previously been overlooked as potential GABAergic sources. For instance, although the superior colliculus (SC) strongly projects to the caudal IO (Weber et al., 1978; Kleinfeld et al., 1999), including the cdMAO (Fujita et al., 2020), it has not yet been considered as a potential inhibitory input to the cdMAO. Likewise, it has also not been explored to what extent the CN may provide a disynaptic inhibition to the cdMAO via one of the more rostral areas in the midbrain.

Here, we investigated these possibilities. We injected a retrograde Cre-dependent tracer in the caudal IO of GAD2-Cre mice and observed GABAergic cells in SC projecting to the contralateral IO. We then confirmed in a transsynaptic tracing experiment that the MCN and lateral CN (LCN) both provide a contralateral, presumptively excitatory, input to these GABAergic cells in the SC. The SC cells receiving MCN input provide a predominantly inhibitory projection to the contralateral cdMAO, whereas the SC cells receiving LCN input provide a relatively equal inhibitory and excitatory input to the contralateral cdMAO and a virtually exclusive excitatory input to subnucleus c of the contralateral cMAO (ccMAO). Anterograde tracing combined with postembedding GABA-immunocytochemistry at the electron microscopic level confirmed a strong inhibitory component of the projection from SC to contralateral cdMAO. Taken together, our data reveal the source of a GABAergic input to the cdMAO that can be controlled indirectly, in a disynaptic fashion, by the CN. These data highlight the unique character of these projections in terms of laterality, polarity and cross-modular nature, pointing toward a specific role in left-right directional control of oculomotor and upper body movements.

## Materials and Methods

### Animals

For retrograde tracing experiments we used male and female Gad2-Cre mice on a C57Bl/6J background (028867; Jackson Laboratories), and for the anterograde transsynaptic experiments at the light microscopic level as well as for the anterograde tracing experiments at the electron microscopic level, we used male and female wild-type mice on a C57Bl/6J background as well as Gad2-Cre mice on a C57Bl/6J background or DAT-Cre wild-type littermates (006660; Jackson Laboratories).

All mice were 52–200 d old, group-housed and maintained on a 12 h light/dark cycle with ad libitum food and water. All experimental procedures were approved a priori by an independent animal ethical committee (DEC-Consult) as required by the relevant institutional regulations of the Erasmus MC and the Dutch legislation on animal experimentation. Permission was filed under the license number AVD1010020197846.

### Experimental design

All anatomical experiments contained a minimal number of two animals with successful injections in the targeted region. Upon confirmation of successful injection site by visual inspection of the targeted region and the concurrent brain-wide anterograde or retrograde labeling, mice were included and the data analyzed accordingly. Since our experiments yielded results in which projections were either present or not, no statistical analysis or numerical comparisons were performed in this study. For our injections in the CN, we targeted the coordinates relatively dorsal so as to avoid leakage to ventral areas such as the complex of vestibular nuclei (VN) and parabrachial nuclei. Since leakage on the dorsal side to the cerebellar cortex will not result in any labeling outside of the cerebellum (CN and VN are the sole output of the cerebellar cortex), this leakage of the injection site is unlikely to result in false-positive labeling of brainstem areas in our experiments.

### Tracers

For the retrograde and anterograde light microscopic experiments in the GAD2-Cre mice, we used AAVrg-CAG-FLEX-rc [Jaws-KGC-GFP-ER2] (titer >1 × 10^12^ vg/ml, 84445; Addgene) or AAV9-CAG-FLEX-GFP (titer >1 × 10^12^ vg/ml, AV5220C; UNC vector core), respectively. To optimally visualize the injection site of the retrograde virus, we co-injected cholera toxin subunit B (CTB; mix 1:1, final concentration 0.5%). For the transsynaptic experiments in wild types, we used adeno-associated virus AAV1-CMV-HI-eGFP-Cre-WPRESV40 (titer 8 × 10^12^ vg/ml, 105545-AAV1; Addgene) that expresses Cre and GFP in the nucleus, while applying AAV9-hsyn-DIO-mCherry (>1 × 10^13^ vg/ml, No 50459-AAV9; Addgene) in the SC as a Cre-dependent tracer. For the mesodiencephalic junction (MDJ) tracing experiment, we injected a retrograde Cre virus AAVrg-pAAV-EF1a-Cre (titer >1 × 10^12^, No 55636-AAVrg; Addgene) in IO and a Cre-dependent virus pAAV9-EF1a-double floxed-hChR2(H134R)-EYFP-WPRE-HGHpA (titer >1 × 10^13^, No 20298-AAV9; Addgene) in MDJ, labeling MDJ fibers in IO. For the anterograde tracing at the electron microscopic level, we used WGA-HRP (L3892; Sigma-Aldrich), which was diluted to 7% in saline, or pAAV9-hSyn-eGFP (titer >1 × 10^12^, 50465-AAV9; Addgene).

### Stereotactic surgeries

For the retrograde and anterograde experiments in GAD2-Cre mice 20–30 nl pAAV-CAG-FLEX-rc was co-injected with CTB (0.5%) in the cdMAO (L −3.2, ML −0.1, DV −5.2), while 60 nl AAV9-FLEX-GFP was injected in the SC (B −4.1, ML +1.5, DV −1.55). For the anterograde transsynaptic experiments investigating the amount of GABAergic varicosities in the IO coming from cells in the SC that also receive cerebellar input, we injected 120 nl of AAV1-Cre (see above) in the CN, aimed at either the MCN (B −6.64, ML −0.7, DV −2, *n* = 2) or the LCN (B −6.05, ML −2.15, DV −2.15, *n* = 2), while we injected 60 nl of Cre-dependent AAV-mCherry (see above) in the SC of the same animals (B −4.1, ML +1.5, DV −1.55). For the electron microscopic investigations of terminals in the IO coming from the SC, we performed injections with WGA-HRP (*n* = 4) or pAAV9-hSyn-eGFP (*n* = 2) aimed at the ventrolateral part of the SC (B −4.0, ML −1.25, DV −1.75). For all intracranial injections, mice were deeply anesthetized with isoflurane (1–2%; Isoflutek, Laboratories Karizoo) and placed in a stereotaxic apparatus (Kopf Instruments). All mice received subcutaneous injections of 50 µg/kg of buprenorphine (Temgesic, Indivior) and 5 mg/kg of Rimadyl (Carprofen, Zoetis), and their eyes were protected from dehydration using DuraTears (Alcon Laboratories). The skull was exposed with a small cutaneous incision and craniotomies were drilled above the IO, CN, or SC, the coordinates of which were obtained from the Paxinos Reference Atlas (Franklin and Paxinos, 1997). Tracer injections were made with pulled glass capillaries (tip outer diameter, ∼15 µm; Hirschmann ringcaps). Following insertion at 500 µm/min, the capillary was left in place for 5 min before pressure injection of the tracer at 5–25 nl/min. To reduce backflow into the pipette tract, the capillary was kept in place for at least 10 min to allow for diffusion of the tracer in surrounding tissue and was then retracted at 100 µm/min. After injections, the skin was closed using Histoacryl skin glue. The mice were returned to their home cage individually and monitored for at least 45–60 min while recovering on a heating pad.

### Processing of material for light microscopy

Following transcardial perfusion with 4% paraformaldehyde (PFA) 14–28 d after viral injection, 3 d for WGA-HRP, the brains were dissected and postfixed in 4% PFA for 1.5 h at room temperature (RT). Subsequently, the brains were transferred to 10% sucrose in 0.1 M phosphate buffer (PB) and left overnight at 4°C, before embedding in gelatin. The brains were incubated in 14% gelatin/10% sucrose in 0.1 M PB for 30 min at 37°C, embedded in a plastic mold, and left at 4°C for at least 30 min to harden. Brains were then cut in small blocks and placed in a 10% formalin/30% sucrose in 0.1 M PB for at least 2.5 h at RT. Embedded brains were transferred to 30% sucrose in 0.1 M PB overnight at 4°C before being sliced on a Leica SM2000R microtome (Leica Biosystems) at 50 µm. To prepare the slices for free floating antibody staining, they were blocked for 1 h with 10% normal horse serum (NHS) and 0.5% triton in PBS at RT after rinsing in PBS. Subsequently, for fluorescence microscopy, slices were stained for GAD67 with mouse anti-GAD67 (MAB5406; 1:2,000, Millipore), for VGLuT2 with guinea-pig anti-VGLuT2 (AB2251-I; 1:2,000, Millipore), for CTB with goat anti-CTB (703; 1:2,000, List Labs), and for GFP with chicken anti-GFP (GFP-1020; 1:2,000, Aves) in 2% NHS and 0.4% triton in PBS and left 24 h at RT. Next, slices were incubated for 2 h at RT in 2% NHS and 0.4% triton in PBS containing anti-chicken-AF488, anti-mouse-cy5, anti-goat-cy3, or anti-guinea-pig-AF405 secondary antibodies (703-545-155, 715-175-150, 705-165-147, or 706-475-148, respectively; Jackson ImmunoResearch Europe Ltd.). Slices were rinsed for 40 min before being mounted onto cover slides (VWR) or glass slides with Mowiol (Carl Roth) or aqueous mounting media.

### Processing of material for electron microscopy

The four animals injected with WGA-HRP were anesthetized with pentobarbital and perfused transcardially with 15 ml 0.9% saline followed by 75 ml of 5% glutaraldehyde in 0.9% saline and 0.18 M sodium cacodylate buffer following 3 d survival time. The brains were placed at 4°C for 1 h and subsequently the brainstems including the IO and SC were cut in 80-mm-thick sections on a vibratome. The WGA-HRP was visualized with TMB and stabilized with DAB-Cobalt (De Zeeuw et al., 1988). After 15 min incubation in 8% glucose and 0.1 M sodium cacodylate, the brains underwent osmification for 90 min in 2% osmium tetroxide and 8% glucose in 0.1 M sodium cacodylate, except for one brain to which we added 1.5% ferricyanide in 0.1 M sodium cacodylate. Subsequently, all sections containing the IO were rinsed with MilliQ (from the Direct 16 machine by Millipore) and dehydrated with ethanol and propylene oxide before embedding in Durcupan. Some sections were stained en block for 90 min in 4% neodymium(III) acetate hydrate and dehydrated with ethanol and 2,2-dimethoxypropane before the embedding in Durcupan.

The two mice with viral injections were anaesthetized with pentobarbital following a survival time of 21 d and perfused transcardially with 15 ml 0.9% saline followed by either 75 ml of 2% glutaraldehyde, 2% PFA and 0.12 M sodium cacodylate, or 75 ml of 4% PFA and 0.5% glutaraldehyde in 0.12 M sodium cacodylate. Specimens were left overnight at 4°C in the same fixative, before cutting in 80 µm slices on a vibratome. Slices containing the IO were blocked for 1 h in 10% Normal horse serum in PBS. Thereafter, for visualization of GFP slices were incubated with 2% NHS/rabbit-anti-GFP (1:5,000, Abcam 290) in PBS for three nights, followed by two nights incubation in 2% NHS/biotinylated goat-anti-rabbit (1:200, BA-1000, Vector) before incubation in ABC (PK6100, Vector) in PBS for two nights. All incubations were performed at 4°C on a shaker. Subsequently, to detect the GFP at the ultrastructural level, a DAB staining was performed, followed by a 15 min incubation in 8% Glucose in 0.1 M sodium cacodylate prior to a 90 min osmication step with 2% osmium tetroxide/8% glucose/1.5% ferricyanide in 0.1 M sodium cacodylate. Finally, all sections were rinsed with MilliQ and stained en block for 90 min in 4% neodymium(III) acetate hydrate and dehydrated with ethanol and 2,2-dimethoxypropane before embedding in Durcupan.

Areas of the contralateral caudal IO containing anterograde labeling were selected and cut into semi-thin sections to verify labeling. Subsequently, ultrathin sections of 50 nm were cut and placed on formvar-coated nickel grids. To visualize inhibitory terminals in the caudal IO, post-embedding GABA immunocytochemistry was performed. The grids were washed twice with MilliQ, and placed for 10 min in 0.05 M glycine. Sections were then rinsed twice in 0.05 M Tris buffer (pH 7.6) containing 0.9% NaCl and 0.1% Tween-20, and left overnight in a droplet of rabbit anti-GABA antibody (A2052; Sigma-Aldrich) diluted 1:1,000 in TBS-T. Subsequently, the grids were rinsed in TBS-T (twice at pH 7.6 and twice at pH 8.2) and incubated for 2 h in a droplet of goat-a-rabbit 10 nm gold (1:30 in TBST pH 8.2). Following this final incubation step, the grids were rinsed twice in TBS-T (pH 7.6) and MilliQ and contrasted with uranyl acetate (10 min) and lead citrate (1 min).

### Imaging and analysis

For all fluorescence experiments, one in every four slices was imaged with a fluorescence Zeiss Axio slide scanner with a 10× objective. Injections were identified by delineating the injection spot on the Paxinos atlas (Franklin and Paxinos, 1997). Additionally, brain-wide projection patterns were compared to the known projections of the intended target nucleus. For the GAD2-Cre fluorescence experiments, presence or absence of cells and fibers was determined by inspection of 10× scans. To quantify GAD67+ and GAD67− varicosities a 350 × 350 µm Z-stack was made, with 2,048 pixels × 2,048 pixels with a 1 airy unit pinhole and optimal z-step size with a 40x objective, using a Zeiss LSM700 confocal microscope.

For the electron microscopy experiments, ultrathin sections containing anterogradely labeled structures (WGA or virus) were studied. A structure was considered WGA-HRP+ or virus+ when one or more crystals or a dark background staining filling the entire structure were present, respectively. For each mouse, we determined the percentage of labeled structures that was also positive for GABA. A structure was determined GABA+ when there were a significantly larger number of gold particles within the structure, compared to surrounding tissue (De Zeeuw et al., 1988). The percentage of double labeled terminals was averaged across all mice. Structures were only counted if the surrounding ultrastructure was well preserved. Data were excluded for analysis when the location of the labeling was unclear.

## Results

### Superior colliculus provides GABAergic input to cdMAO

**Figure 1.**
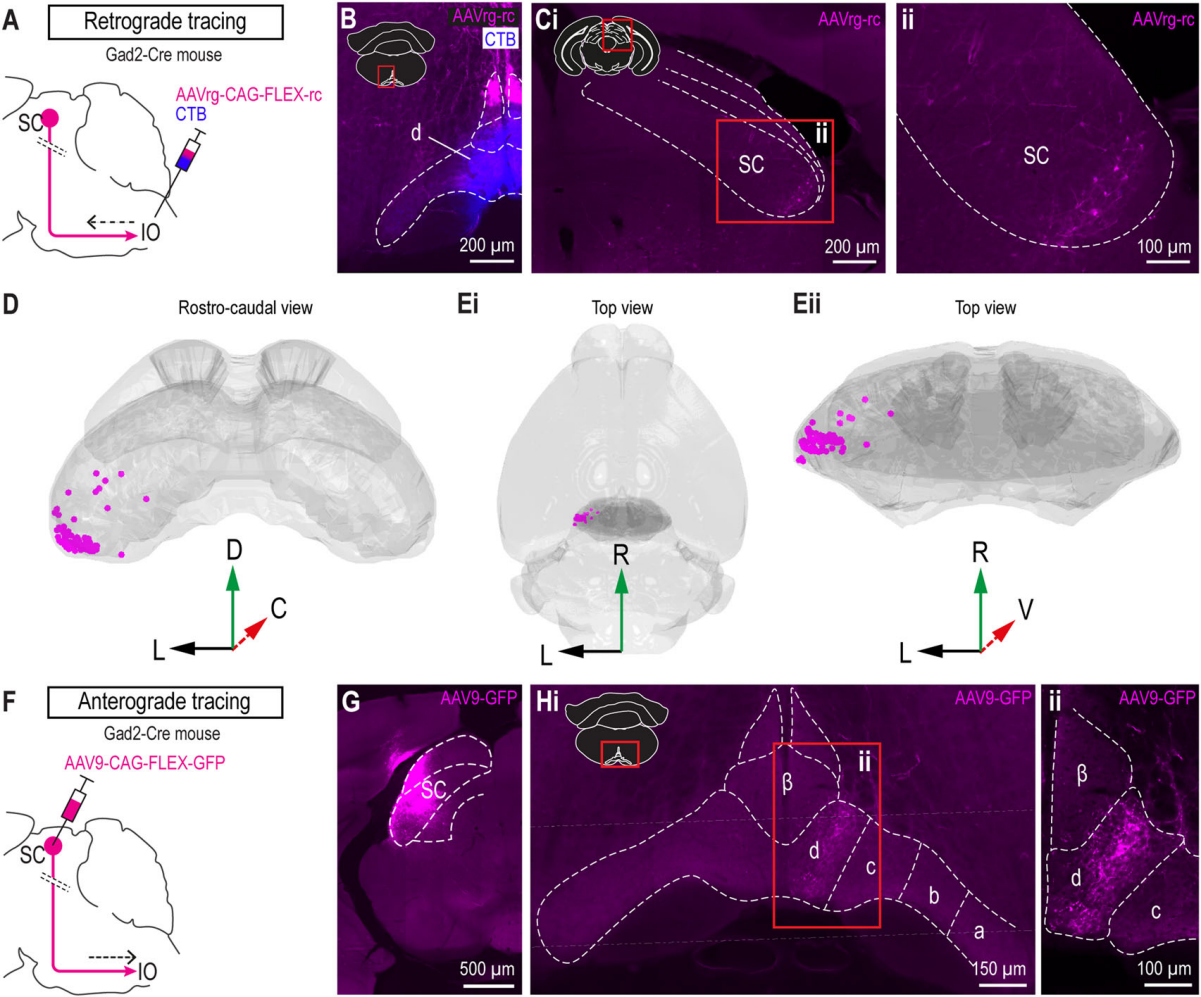
Retrograde and anterograde tracing in GAD2-Cre mice to uncover the inhibitory SC-cdMAO projection. ***A***, Schematic representation of retrograde tracing strategy. Dotted lines represent crossing of SC-IO fibers to the contralateral side. ***B***, Cre-dependent AAVretro-GFP (magenta) and CTB (blue) were simultaneously injected in IO of GAD2-Cre mice (*n* = 2). CTB was co-injected to visualize the injection site. ***C***, Following injections in the IO, retrogradely labeled cells were observed in the lateral SC. Left, overview picture of labeling in SC; right, magnification of labeled cells. ***D,E***, Rostro-caudal (***D***) and dorso-ventral (***E***) 3D plots of retrogradely labeled cells in SC. ***F***, Schematic representation of anterograde tracing strategy. Cre-dependent AAV9 was injected in the lateral SC of Gad2-Cre mice (*n* = 2). ***G***, Representative example of injection spot in SC. ***H***, Subsequent labeling of fibers in the contralateral subnucleus d of the caudal MAO (cdMAO) at low (***Hi***) and high (***Hii***, corresponding to inset in ***Hi***) magnification. No significant labeling was observed in other IO subnuclei.

**Figure 2.**
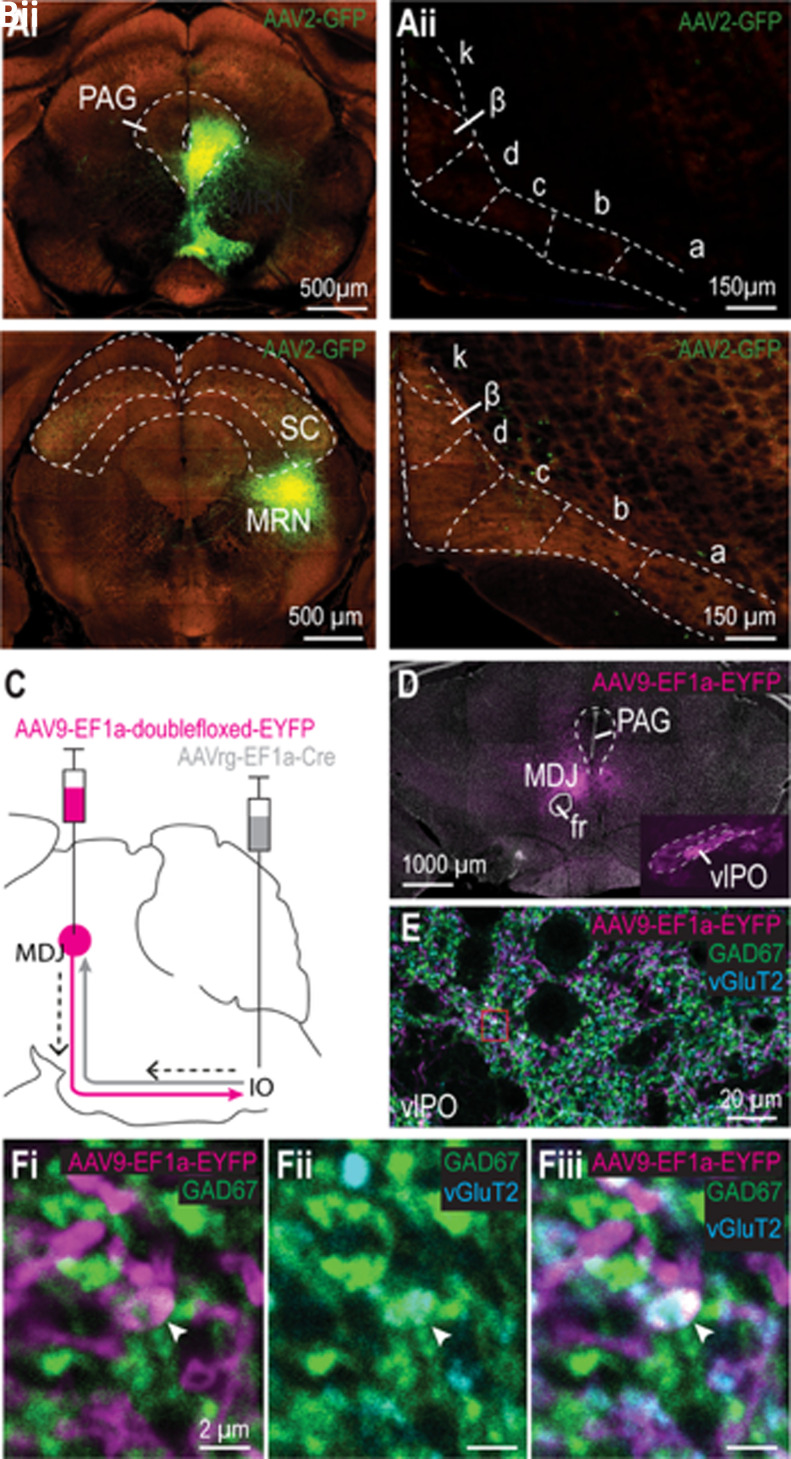
Anterograde investigation of projections from PAG, MRN and MDJ to cdMAO. ***A*,*B***, Representative examples of injections restricted to PAG (***Ai***) and MRN (***Bi***) and the absence of labeled varicosities in the cdMAO following these injections (***Aii*,*Bii***). Data obtained from the Allen Mouse Brain Atlas. ***C***, Schematic representation of injections for investigation of MDJ-IO projection. ***D***, Representative example image of cells expressing Cre-dependent tracer in MDJ cells (positioned around the fasciculus retroflexus (fr) where the fr crossed the medial lemniscus along the dorsoventral axis) that project to IO (retrograde Cre virus injected in IO) and the resulting anterograde labeling in the IO (***D***, inset). ***E***, Anterogradely labeled MDJ fibers in vlPO in slices that are co-stained for GAD65/67 and VGLuT2. ***F***, Single Z-slice of an exceptional MDJ varicosity that encompasses both GAD65/67 and VGLuT2.

To investigate which brain areas may provide inhibitory input to the cdMAO we co-injected CTB with a retrograde Cre-dependent vector in the caudal IO of GAD2-Cre mice (Fig. 1*A,B*), thereby labeling GABAergic cells that project to the caudal MAO. CTB staining confirmed that the injection was centered around the cdMAO, as identified by the precise location of the injection spot, which can be ambiguous based on a retrograde viral tracer alone (Fig. 1*B*). In the mesencephalon we found prominent retrograde labeling in the SC, specifically in the ventrolateral intermediate grey layer (InG) (Fig. 1*C–E*). Additionally, we observed some scattered cells in the midbrain reticular nucleus (MRN; i.e., the reticular area surrounding the SC), the periaqueductal grey (PAG), and the MDJ. For all of these retrogradely labeled areas, we did and/or analyzed anterograde tracing experiments to either prove or disprove a possible inhibitory projection to the caudal MAO, specifically to the cdMAO. For all areas, except the SC (Fig. 1*F*–*H*), we could not confirm a convincing inhibitory projection to cdMAO (Fig. 2). More specifically, based on injections from the Allen Brain Institute, the PAG (Fig. 2*A*) and MRN (Fig. 2*B*) did not provide a significant projection to cdMAO, and MDJ projections to the IO, identified in our anterograde tracing experiment (Fig. 2*C*,*D*), appeared to be virtually completely glutamatergic (VGLuT2+) with a very sparse (<1%) occurrence of varicosities that co-expressed VGLuT2+ and GAD65/67+ (Fig. 2*E*). In contrast, upon injecting an anterograde Cre-dependent tracer in the ventrolateral part of InG of the SC in GAD2-Cre mice (Fig. 1*F*,*G*), we observed a strong GABAergic projection targeting the cdMAO specifically (Fig. 1*H*). Together, these tracing results suggest that the SC is the only source of GABAergic input to the cdMAO.

### MCN and LCN provide disynaptic projection to ipsilateral cdMAO via contralateral SC

**Figure 3.**
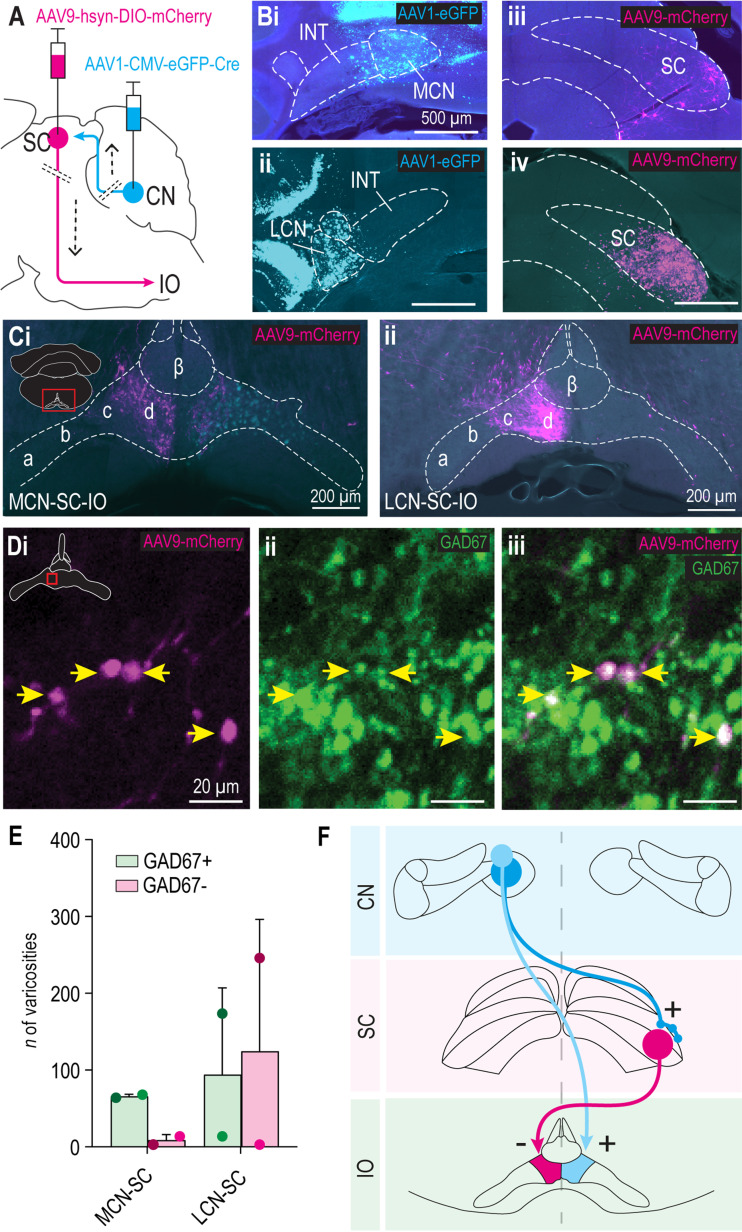
Anterograde transsynaptic tracing from MCN and LCN to IO via SC. ***A***, Schematic representation of anterograde transsynaptic tracing strategy. ***B***, Transsynaptic AAV1 expressing Cre and eGFP (cyan) was injected in the MCN (*n* = 2) or LCN (*n* = 2), followed by an injection of Cre-dependent mCherry (magenta) in the lateral part of SC (all animals). ***Bi*–*iv***, Representative example photos of injection spots in MCN (***Bi***) and LCN (***Bii***) and subsequent transsynaptic labeling of cells in SC following injection in MCN (***Biii***) and LCN (***Biv***). Note that a small leakage to the interposed nucleus (INT) cannot be excluded in ***Bi*** and ***Bii***, but the MCN and LCN injections never contaminated each other. ***C***, Anterograde labeling in the IO following transsynaptic tracing; the labeling is predominantly ipsilateral to the MCN (***Ci***) and LCN (***Cii***) that were injected. Note that some transsynaptically labeled cells can be observed in the IO contralateral to MCN injection spot (***Ci***). ***D***, Images of a fiber in the caudal MAO with varicosities (yellow arrows) positive for mCherry (***Di***), GAD67 (***Dii***), or both (***Diii***). ***E***, Number of GAD67+ and GAD67− varicosities in cdMAO for MCN and LCN injections. Error bars represent standard deviation (SD), and dots represent number of varicosities in individual mice. ***F***, Summary panel demonstrating the laterality of the observed inhibitory disynaptic projection from the MCN to the cdMAO and the complementary direct excitatory projection from the MCN.

Given the fact that only the SC was confirmed to provide a GABAergic input to the cdMAO (Figs. 1, 2), we set out to investigate whether the CN target the inhibitory neurons in the SC that project to the cdMAO. We focused on the MCN and the LCN, which can be readily separated from each other (Fig. 3). Moreover, the MCN is part of the olivocerebellar module in which the cdMAO participates (Apps and Hawkes, 2009; De Zeeuw and Ten Brinke, 2015), and the LCN has been shown to also provide a strong projection to SC (Teune et al., 2000). Consequently, we injected MCN and LCN with AAV1-Cre and the SC with Cre-dependent AAV9-DIO-mCherry (Fig. 3*A*), enabling us to trace projections to IO. Indeed, the transsynaptically labeled cells in the SC showed a clear projection to the contralateral cMAO for both MCN and LCN experiments (Fig. 3*B–E*). Interestingly, because the CN target the contralateral SC and the SC targets the contralateral IO, the disynaptic projection from CN to IO ends up in the cdMAO ipsilateral to the “starting” CN, effectively transferring inhibition to the ipsilateral olivary neurons (Fig. 3*F*). The disynaptic projection from the MCN via the SC terminated in the cdMAO specifically (Fig. 3*C*), with a striking absence of fibers in the ccMAO, which is in line with the GABAergic projection pattern from SC to cdMAO in the GAD2-Cre mice (Fig. 1*H*). Most (i.e., 88%) of these terminals in the cdMAO were GAD67+ (Fig. 3*D,E*). In contrast, the projection starting from the LCN was spatially less restricted, as its fibers also extended into the neighbouring ccMAO (Fig. 3*C*). The GAD67+ proportion of the transsynaptically labeled fibers in the cdMAO from the LCN was approximately half (Fig. 3*E*), whereas virtually none of the transsynaptically labeled varicosities in the ccMAO were GAD67+. The GABAergic and somewhat larger nonGABAergic neurons of the SC that received input from the LCN were located to a large extent in the same location as the GABAergic neurons that received input from the MCN, i.e., in the ventrolateral InG of the SC. Taken together, the SC neurons receiving input from the MCN appear to have a rather prominent and specific GABAergic projection to the cdMAO, not extending into the surrounding IO subnuclei (Fig. 3*F*) and thereby mirroring the direct excitatory projection from MCN to cdMAO that comes from the other side (Wang et al., 2023). In contrast, LCN cells target both the GABAergic pathway from SC to cdMAO and a parallel nonGABAergic, potentially excitatory, pathway from SC to cdMAO and ccMAO.

### Ultrastructural analysis of SC projection to cdMAO

**Figure 4.**
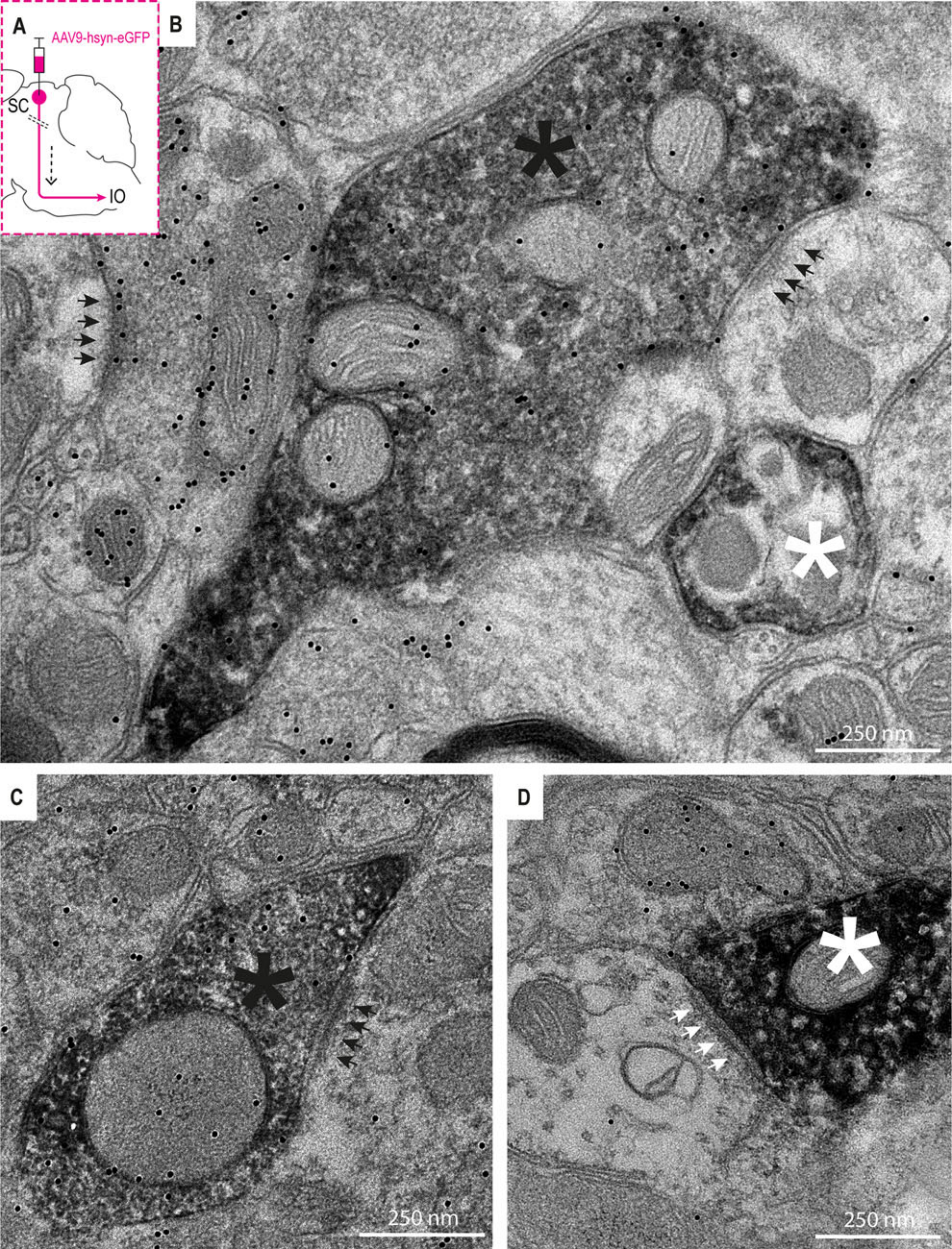
Electron microscopy confirms the presence of SC-originating GABAergic terminals in contralateral cdMAO. ***A***, Schematic overview of experiment; we injected AAV-GFP in the superior colliculus and investigated the contralateral cdMAO. ***B***, Electron micrograph showing a GABAergic terminal (black asterisk) in the contralateral cdMAO; the GABAergic nature of the terminal is demonstrated by the immuno-gold particles (black dots). Synapses of GABAergic terminals onto olivary spines are indicated by black arrows. A similar AAV-GFP labeled terminal, yet nonGABAergic, is indicated by white asterisk. ***C***, Same as in ***B***, but now a GABAergic SC terminal contacting a dendrite. ***D***, AAV-GFP labeled terminal that is nonGABAergic (white asterisks) forming a synapse (white arrows) onto a dendrite.

To further investigate the GABAergic component within the projection from SC to cdMAO at the ultrastructural level, we injected the SC either with AAV9-hSyn-eGFP (*n* = 2) or WGA-HRP (*n* = 4). As we did not observe differences in the injection sites or projection patterns, the results of these mono-synaptic experiments are described together. For both the AAV-GFP and WGA-HRP experiments, the injection site encompassed the SC and surrounding regions, including the MRN. Labeled fibers and varicosities were observed predominantly contralaterally in the cdMAO and ccMAO. We found numerous single and double labeled profiles in the contralateral cdMAO under the electron microscope; these included single labeled AAV-GFP, WGA-HRP and GABA labeled terminals, preterminal segments and myelinated axons as well as double labeled AAV-GFP-GABA and WGA-HRP-GABA terminals, preterminal segments and myelinated axons (Fig. 4*A–C*). In line with our light microscopic data (Figs. 1–3), 52% of the AAV-GFP labeled structures and 50% of the WGA-HRP labeled structures were GABA-positive. Many of the single and double labeled terminals made synaptic contacts with GABA-negative dendrites or spines, showing the asymmetric or symmetric configuration typical of excitatory or inhibitory synapses, respectively.

## Discussion

**Figure 5.**
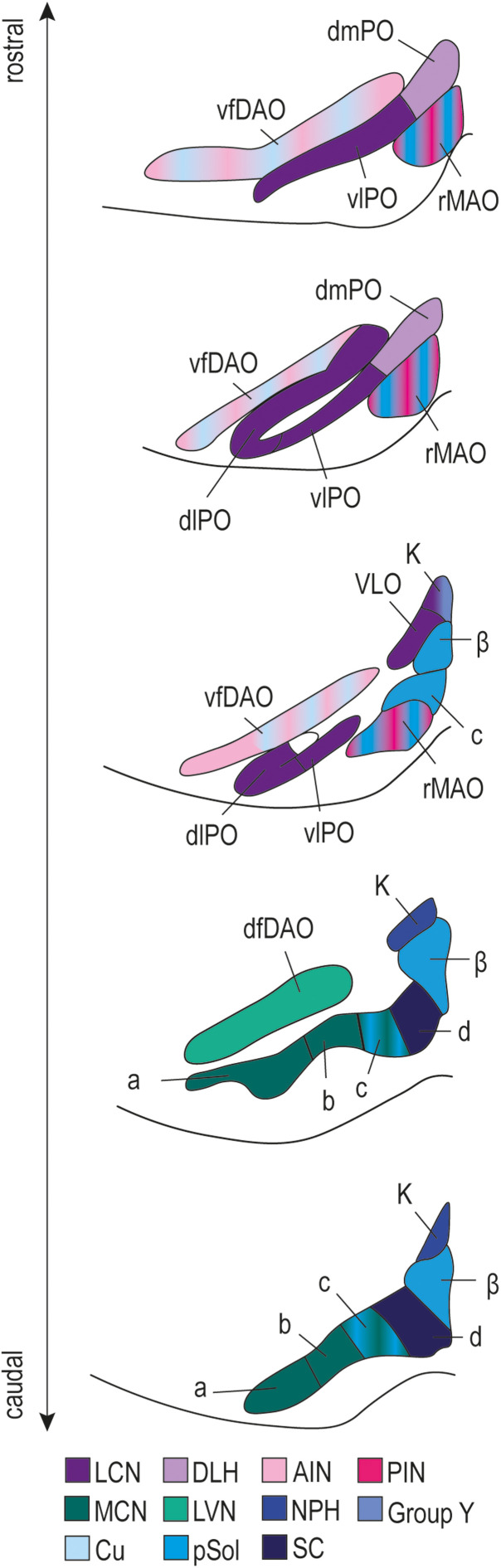
Map of inhibitory inputs to the subnuclei of the IO. The map with all known inhibitory projections to the IO presents a state-of-the-art overview in which all investigations for all mammalian species have been combined; these include studies in mice, cats, rats and rabbits (for details see Table 1). The scheme is depicted in caudo-rostral direction (bottom to top) where all inhibitory inputs are represented in color (for coding see insets at the bottom). All projections are contralateral, except for that of the NPH, which is bilateral, and that of the pSol, which is ipsilateral. Note that all olivary subnuclei have now at least one known source of inhibition. The subpanels containing the cdMAO represent the IO from Bregma −7.76 mm to −7.56 mm (Paxinos Atlas for mice). Abbreviations in alphabetical order: a, subnucleus a of the caudal medial accessory olive; AIN, anterior interposed nucleus; b, subnucleus b of the caudal medial accessory olive; β, nucleus beta; c, subnucleus c of the caudal medial accessory olive; Cu, cuneate nucleus; d, subnucleus d of the caudal medial accessory olive; DAO, dorsal accessory olive; DLH, dorsolateral hump; DLP, dorsolateral protrusion of the medial cerebellar nucleus; dlPO, dorsal leaf of principal olive; dmPO, dorsomedial principal olive; K, dorsal cap of Kooij; LVN, lateral vestibular nucleus; MCN, medial cerebellar nucleus; LCN, lateral cerebellar nucleus; NPH, nucleus prehypoglossus; PIN, posterior interposed nucleus; pSol, parasolitary nucleus; rMAO, rostral medial accessory olive; SC, superior colliculus; VLO, ventrolateral outgrowth of principal olive; vlPO, ventral leaf of principal olive.

**Figure 6.**
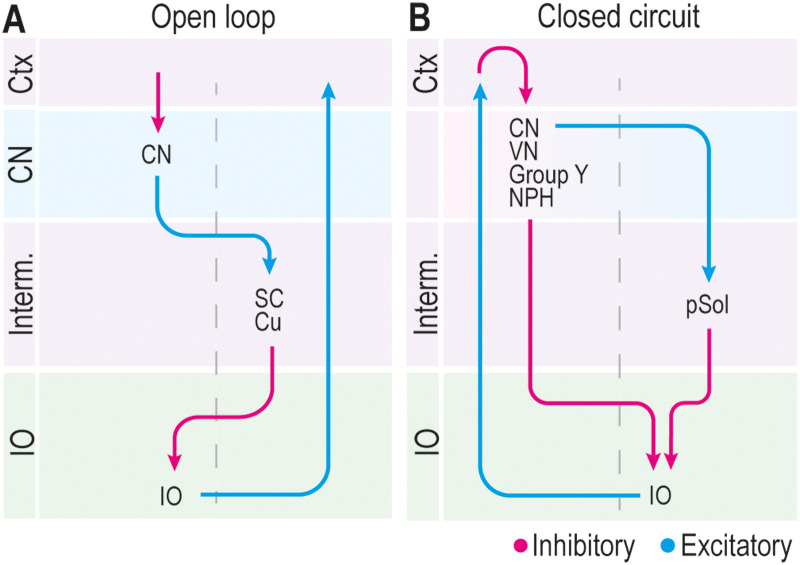
Open loops and closed circuits of olivocerebellar modules. ***A***, Only two disynaptic inhibitory projections appear to be open-loop modules; these projections emerge from one side of the cerebellar cortex (Ctx) and nuclei (CN) and project to the contralateral superior colliculus (SC) or cuneate nucleus (Cu), ending up in the inferior olive (IO) on the same side as where the Ctx and CN projections start. We refer to this projection as open loop, because the climbing fibers derived from the IO cells do not project back to the same side of the Ctx but the opposite side. ***B***, All other olivocerebellar modules appear to function as closed circuits in that there are inhibitory projections from the CN or vestibular complex, including nucleus prepositus hypoglossi (NPH), Group Y, lateral vestibular nucleus (LVN) as well as parasolitary nucleus (pSol), to the contralateral IO (De Zeeuw et al., 1994b), and that here the climbing fibers project back to the Purkinje cells in the Ctx on the same side as where the olivocerebellar modules originates. Note that the inhibitory projection from pSol to the IO via the CN/VN complex in the closed circuit network is also disynaptic, similar to the CN-SC-IO projection in the open loop situation.

In the current study, we demonstrate a disynaptic inhibitory projection from the MCN to the cdMAO of the caudal IO via the SC at both the light microscopic and electron microscopic level. This is the first description of an inhibitory pathway to the cdMAO to date. Together with previous reports, this finding completes the map of inhibitory inputs to IO subnuclei in that we now have at least one source of inhibition for all IO subnuclei (Fig. 5). The laterality of this disynaptic MCN-SC-IO inhibitory projection, which is in effect ipsilateral, appears rather unique among the olivocerebellar modules, which typically comprise a contralateral inhibition in a closed circuit from the hindbrain to the various olivary subnuclei (Voogd et al., 2012; Apps et al., 2018). As a result, the MCN-SC-IO connection is integrated in an olivocerebellar module that forms an open loop (Fig. 6), because the climbing fibers derived from the cdMAO are still projecting to Purkinje cells on the contralateral side, just like all other climbing fibers (De Zeeuw and Ten Brinke, 2015). This rather unique neuro-anatomical configuration may point toward specific functionalities for the behaviors involved.

**Table 1.** List of studies with evidence for inhibitory input to the various olivary subnuclei

IO subnucleus	Input IO	Laterality	Publication	Technique	Species
Caudal K	NPH	Bilateral Bilateral	(De Zeeuw et al., 1993) (Arts et al., 2000)	Anterograde tracing + GABA Physiology + EM + GABA	Rat/rabbit Rabbit
Rostral K	Dorsal group y Ventral LCN	Contralateral Contralateral	(De Zeeuw et al., 1994a) (De Zeeuw et al., 1994a)	Anterograde/retrograde tracing + GABA + EM Anterograde/retrograde tracing + GABA + EM	Rabbit Rabbit
VLO	LCN	Contralateral	(De Zeeuw et al., 1994a)	Anterograde/retrograde tracing + GABA + EM	Rabbit
Β	pSol	Ipsilateral	(Barmack et al., 1993) (Barmack et al., 1998) (Barmack and Yakhnitsa, 2000)	Physiology Anterograde and retrograde tracing + GABA Physiology	Rabbit Ratrabbit Rabbit
cMAO (a)	MCN	Contralateral	(Wang et al., 2023)	Anterograde Cre-dependent tracing	Mouse
cMAO (b)	MCN	Contralateral	(Wang et al., 2023)	Anterograde Cre-dependent tracing	Mouse
cMAO (d)	SC	Contralateral	Current paper	Anterograde Cre-dependent + GABA + EM	Mouse
cMAO (c)	pSol MCN	Ipsilateral Contralateral	(Nelson and Mugnaini, 1989) (Wang et al., 2023)	Retrograde tracing + GABA Anterograde Cre-dependent tracing	Rat Mouse
rMAO	pSol	Ipsilateral	(Barmack et al., 1998)	Anterograde and retrograde tracing + GABA	Rat/rabbit
	PIN	Contralateral	(de Zeeuw et al., 1988) (de Zeeuw et al., 1989) (Loyola et al., 2023)	Anterograde tracing + GABA Anterograde tracing + GABA Physiology	Cat Cat Mouse
PO	LCN	Contralateral	(de Zeeuw et al., 1988) (Loyola et al., 2023)	Anterograde tracing + GABA Physiology	Cat Mouse
dmPO	DLH	Contralateral	(de Zeeuw et al., 1988)	Anterograde tracing + GABA	Cat
DAO	Cu	Contralateral	(Nelson and Mugnaini, 1989)	Retrograde tracing + GABA	Rat
	LVN	Contralateral	(Fredette and Mugnaini, 1991)	Lesion study + GABA	Rat

Physiological studies were (generally) less detailed regarding precise targeting of CN or IO subnuclei, as they complemented previous detailed anatomical reports that had described the projections in question in detail. Abbreviations: a, subnucleus a of the caudal medial accessory olive; AIN, anterior interposed nucleus; b, subnucleus b of the caudal medial accessory olive (cMAO); β, nucleus beta; c, subnucleus c of the caudal medial accessory olive; Cu, cuneate nucleus; d, subnucleus d of the caudal medial accessory olive; DAO, dorsal accessory olive; DLH, dorsolateral hump; DLP, dorsolateral protrusion of the medial cerebellar nucleus; dlPO, dorsal leaf of principal olive; dmPO, dorsomedial principal olive; K, kaap van kooij; LVN, lateral vestibular nucleus; MCN, medial cerebellar nucleus; LCN, lateral cerebellar nucleus; NPH, nucleus prehypoglossus; PIN, posterior interposed nucleus; pLCN, parvocellular lateral cerebellar nucleus; pSol, parasolitary nucleus; rMAO, rostral medial accessory olive; SC, superior colliculus; VLO, ventrolateral outgrowth of principal olive; vlPO, ventral leaf of principal olive.

Interestingly, although the direct MCN-cdMAO projection (Wang et al., 2023) follows the modular closed circuit organization of the nucleo-olivary projections (Ruigrok and Voogd, 1990), it comprises excitatory glutamatergic synapses, contrary to the usual GABAergic nucleo-olivary projections. This stresses the relevance of the currently described MCN-SC-IO pathway targeting the ipsilateral cdMAO, hereby complementing the direct excitatory projection from the MCN to the contralateral cdMAO (Wang et al., 2023) with an, in effect, ipsilateral inhibition (Fig. 6*A*). This supports the possibility that the left and right side of the olivocerebellar modules modulate or control the direction of head and upper body movements by coordinating antagonistic effects (Fig. 3*F*). While the MCN targets the inhibitory SC-cdMAO projection cells rather selectively, the LCN innervates both inhibitory and excitatory cells in the SC that project to cdMAO as well as excitatory SC cells that project to ccMAO. Thus by incorporating the possibility of disynaptic pathways routing through areas posterior in the midbrain, we are advancing on unraveling the puzzle of the inhibitory projections to the inferior olivary subnuclei that thus far all originated from the hindbrain (De Zeeuw et al., 1988, 1993, 1994a,b; Nelson et al., 1989). Yet, having discovered now at least one source of GABAergic input to all olivary subnuclei does not mean that we have solved the puzzle completely. For example, based on the current study in which we focused on unraveling the indirect GABAergic input from the MCN to the cdMAO via the SC, we cannot exclude the possibility that the PIN, just like the LCN, also contributes to a minor part of the inhibition to the cdMAO via the SC (Fig. 3*B*).

In terms of anatomical connectivity, our findings on the disynaptic projections from the MCN and LCN to the caudal MAO via the ventrolateral InG of the SC agree with those described previously on the CN-SC projections (Teune et al., 2000; Fujita et al., 2020; for review see Novello et al., 2022) and the SC-IO projections (Weber et al., 1978; Akaike, 1986; Benavidez et al., 2021). The CN-SC projections are probably excitatory, because we did not find inhibitory synapses in the SC in the current study, Person and colleagues did not find long-range inhibitory projections from the CN to the SC (Judd et al., 2021), and physiological investigations on the CN-SC projection point toward an excitatory nature (Westby et al., 1993). Likewise, in line with the physiological finding that SC stimulation can result in short-latency climbing fiber responses (Akaike, 1986), our morphological findings confirm that a strong glutamatergic excitatory projection from SC to IO exists, but that this projection is most likely anatomically intermingled with the SC cells that form the inhibitory hub between the MCN and cdMAO. In this respect, it is of relevance to note that simultaneous stimulation of both a glutamatergic and GABAergic projection to IO neurons can still elicit an action potential and that maximum inhibition occurs in a relatively narrow time interval in which the excitation occurs shortly (≍50 ms) after inhibition (Loyola et al., 2023).

It will be interesting to find out which other regions project to cells in the SC that receive a cerebellar input and project to the IO. Areas that come to mind include the primary motor cortex and the dorsal anterior cingulate area, both of which target different parts of the SC (Benavidez et al., 2021). Indeed, it should be elucidated to what extent these cerebral cortical relays via the SC target preferentially the inhibitory or excitatory neurons in the SC. For example, some of the cerebral cortical projections to the lateral SC may well target the glutamatergic cells that project to the ccMAO, possibly to align the SC sensorimotor networks with higher-order associative areas (Benavidez et al., 2021).

One of the main novelties of our findings lies in the fact that the inhibitory cells in the SC that target the cdMAO receive a prominent input from the presumptively excitatory MCN cells, whereas the excitatory cells in the SC that target the cdMAO hardly receive any MCN input (Fig. 3). This targeting is especially remarkable as we could not spatially separate the GABAergic and nonGABAergic cells in the SC that project to IO. Indeed, the GABAergic cells in the ventrolateral InG of the SC that receive MCN input were to a large extent in the same location as the larger nonGABAergic, presumptively glutamatergic (Akaike, 1986), cells that receive an input from the LCN and that do project to the caudal MAO. These findings suggest that the MCN has a way to specifically connect with the GABAergic cells in the SC that project to the cdMAO. How the specificity of this connection evolves during development and which molecular and/or physiological mechanisms support this process form interesting topics for future studies.

What is the function of the inhibitory projection from the MCN to the cdMAO via the SC? The deep layers of the SC play a vital role in specific forms of sensorimotor integration, such as saccade adaptation and gaze control (Grossberg et al., 1997; Catz et al., 2005; Masullo et al., 2019; Zahler et al., 2021). For example, during the generation of goal-directed saccades, the SC may serve as a comparator that computes a mismatch of predictive motor commands and sensory feedback signals (Bergeron et al., 2003). If such computations increase activation of the GABAergic neurons in the InG of the SC, the activity of the olivary neurons downstream in the cdMAO neurons will decrease and thereby facilitate long-term potentiation in the Purkinje cells of the oculomotor vermis due to a decrease in climbing fiber activity (Herzfeld et al., 2015; Gutierrez-Castellanos et al., 2017). Given that the climbing fiber signals in the oculomotor vermis encode direction of error, the potentiation of the Purkinje cells in this area will increase the simple spike activity and thereby shift the ipsilateral eye and corresponding gaze into the temporal direction (Herzfeld et al., 2015). These data are consistent with the finding that stimulating the direct excitatory projection from the MCN to the contralateral cdMAO (see also Fig. 3*F*) induces a gaze shift into the nasal direction by increasing the climbing fiber activity (Wang et al., 2023). Thus, the left and right MCN can work together, synergistically controlling the eye movements and upper body movements both real-time and long-term, such that gaze control via the SC and cdMAO is optimal.

Interestingly, the inhibition provided by the cuneate nucleus to the dorsal accessory olive (DAO), which is controlling upper limb, shoulder, and trunk movements (McCurdy et al., 1998), follows the same configuration as the SC-cdMAO pathway in that it is also an open loop system and following a laterality that is opposite to that of the classical olivocerebellar modules with a closed circuit network (Fig. 6). Moreover, both the SC and the cuneate nucleus receive a prominent input from the contralateral MCN (Teune et al., 2000). Possibly, both open loop systems facilitate control of converging movements that require fast coordination, because of their kinematics with relatively strong high-velocity components. Thus, while the medial cerebellum may implement an internal model to drive firing of premotor neurons of proximal musculature, the inhibitory SC-IO and cuneate-IO neurons may integrate related sensorimotor information and gate the excitability of corresponding olivary neurons in the MAO and DAO, respectively.

Question remains what the role is of the disynaptic pathways to the cMAO controlled by the LCN. The fact that both LCN and MCN appear to exert control over the cdMAO confirms that cross-modular projections exist when an extra synapse is considered. In contrast to the MCN-SC-IO pathway, the LCN-SC-IO pathway provides a mixed inhibitory and excitatory input to cdMAO and an excitatory input to ccMAO. Since LCN sends more widespread projections to not only the SC but also to the various thalamic nuclei, the LCN-SC-IO pathway could be related to active exploration in a complex setting such as a demanding social context (Solie et al., 2022), setting it apart from the “relatively simple” reflex-like control of eye and head movements that is mediated by the MCN-SC-IO pathway. Possibly, the LCN-SC-IO pathway is engaged during an active process of selective attention, steering eye and head movements based upon subtle higher-order cues in the social or physical environment.

## Synthesis

Reviewing Editor: David Schoppik, New York University Decisions are customarily a result of the Reviewing Editor and the peer reviewers coming together and discussing their recommendations until a consensus is reached. When revisions are invited, a fact-based synthesis statement explaining their decision and outlining what is needed to prepare a revision will be listed below. The following reviewer(s) agreed to reveal their identity: Izumi Sugihara, Taro Ishikawa. Note: If this manuscript was transferred from JNeurosci and a decision was made to accept the manuscript without peer review, a brief statement to this effect will instead be what is listed below.

Major comments:

(1)

The GABAergic inhibitory projection was initially found as the nucleoolivary projection or the projection of GABAergic neurons in the cerebellar nuclei. The early paper by Ruigrok and Voogd (1990, why this seminal paper is not cited?) with PHA-L tracing topographic projection from various areas in the CN to various areas in the IO. In this study, they proposed that the topographic relationship of the GABAergic nucleoolivary projection covers the entire cerebellar nuclei and the entire inferior olive nucleus, except for some exceptional parts such as the Dorsal cap of Kooy of the inferior olive. This idea seems to remain as a "Closed circuit" in Figure 6. However, the GABAergic projection from the super colliculus (SC) to the cdMAO belongs to the "Open circuit" in Figure 6. One of the problems of this manuscript is whether the cdMAO has the closed circuit type GABAergic projection from the MN is not examined or even mentioned besides the newly discovered GABAergic projection from the SC.

Indeed, either case R127 or R128 of Ruigrok and Voogd, 1990, in which PHA-L is injected into the caudal and dorsal MCN, seems to be labeled axonal terminals in the cdMAO. These projections should be mentioned and compared with the data of the present study. Ruigrok and Voogd, 1990, also describe that the density of PHA-L-labelled terminals within the caudal MAO, group "beta", VLO and DMCC, and dorsal fold of the DAO was always lower compared to the heavy, massed projections observed within the rostral MAO, PO, DAO, and DM. This description may be noted in the present study.

Therefore, it should be studied (or explained) whether the cdMAO has the closed circuit GABAergic projection from a certain area of the contralateral MAO. If it does not have such a connection, then the meaning of such inconsistent organization of the nucleoolivary projection should be discussed.

Concerning the brain stem input to the caudomedial MAO, the projection from the SC to the MAO shown by Akaike (1986) matches or overlaps the projection by the present study. He seems to think that this projection is excitatory because the SC stimulation evokes short-latency climbing fiber firing in the medial crus II (Akaike, 1986). The present manuscript also shows non-GABAergic projection from the SC to the cdMAO (Fig. 4A, white asterisk). Which are predominant, excitatory, or inhibitory projections from the SC to the cdMAO? How do the authors explain the differences between Akaike (1986) and the present findings? Would there be other major excitatory projections to the cdMAO besides that from the SC?

(2) The cdMAO is the essential subarea of the IO in this study.

The definition of the cdMAO should be clearly described together with the spatial extent of the cdMAO.

In Figures, the presence of the GABAergic terminals in the cdMAO is shown only in a few coronal sections. The entire extent (rostrocaudal extent) of the cdMAO should also be shown (in the horizontal trajectory of the IO, for example, c.f. Figure 9 of De Zeeuw et al., 1996, Morphological correlates of bilateral....., the MAO part of Fig. 2 of Ruigrok and Voogd, 1990; Figure 14 of Akaike, 1986 and other papers).

It is also necessary to identify the cdMAO by indicating its cortical and nuclear target areas. Fujita et al. (2020) have indicated that the cdMAO project to the zebrin-positive areas in vermal lobule VII and medial paravermis in simple lobule, crus I, crus II, and paramedian lobule and the caudal dorsal part of the MCN by referring to some preceding studies. Do the authors agree with this?

(3) Figure 2F-H is missing.

(4) Lines 94-96: "We then confirmed in a transsynaptic tracing experiment that the MCN and lateral CN (LCN) both provide contralateral excitatory input to these GABAergic cells in the SC." On a related note, the projection from the cerebellar nuclei to the superior colliculus is shown as excitatory in Figures 3F and 6A. However, I could not find any data in this manuscript to support these descriptions.

(5) Lines 248-249: Details of "our anterograde tracing experiment" should be explained, as there is no explanation in the Methods and Legend.

Minor Comments

Line 75: "all olivary dendrites of all olivary neurons" sounds redundant.

Line 189, define "MilliQ".

Lines 129-130 and 140: The virus "pAAV-CAG-FLEX-rc" should be written in full without truncation. Its serotype should also be given.

Line 132: The abbreviation "CTB" should be spelled out the first time it appears. The marker conjugated to CTB should be specified.

Line 253-254, "... input to the cdMAO from the hindbrain". Isn't this phrase awkward because the superior colliculus is in the midbrain?

Figure 1 and other figures: Insert space between digits and "micrometer", "200um" should read "200 um".

Figure 1B, Why is the bilateral dorsal cap nuclei of the inferior olive (dorsomedial side) labeled in magenta?

Figure 1D, E: These 3D models are very difficult to understand. Can they be revised somehow?

Figure 1H, How subareas d, c, b, and a were defined in the caudal MAO?

Figure 2: For better understanding, schematic panels (like Figures 1A, 1F and 3A) should be added for each type of experiment.

Figure 2C, "fr" does not seem to be defined. Also, details of "retrograde Cre-expressing virus" should be provided.

Lines 279-281. The author says the LCN project to the SC. As far as I know, the ventral PIN also projects to the SC (Y Kurimoto, S Kawaguchi, M Murata, 1995, Cerebellotectal projection in the rat: anterograde and retrograde WGA-HRP study of individual cerebellar nuclei, Neurosci Res 22(1):57-71. doi: 10.1016/0168-0102(95)00874-s.; G W Westby, C Collinson, P Dean, 1993, Excitatory drive from deep cerebellar neurons to the superior colliculus in the rat: an electrophysiological mapping study Eur J Neurosci 5(10):1378-88. doi: 10.1111/j.1460-9568.1993.tb00924.x.; and other papers). The authors should also look at the ventral PIN as well as the LCN.

Figure 3Bi, ii: Some cortical areas are much more densely labeled than the MCN or LCN. Does not this interfere with the authors' interpretation?

Figure 4. The authors say A and B are symmetric and C is asymmetric. However, I cannot judge as they say. I do not think in these panels, in which the presynaptic component is filled with black reaction product,

Figure 4. The calibration bar is missing.

Line 321-322. "the postembedding GABA-labelling ... is significantly more than..." The meaning is not clear. "more" means "denser"? However, it seems to me that

Figure 5, "DAO" may be more specified to either "ventral fold" or "dorsal fold", which would match with projection differences.

Line 381. "involved control" should read "involved in control" (?)

Line 382: The phrase "a push-pull fashion" is vague. Perhaps more precise terms such as "synergistic" or "coordination of antagonistic effects" should be used. The same phrase in the abstract should also be reconsidered.

## Author Response

Synthesis Statement for Author (Required):

Major comments:

(1)

The GABAergic inhibitory projection was initially found as the nucleoolivary projection or the projection of GABAergic neurons in the cerebellar nuclei. The early paper by Ruigrok and Voogd (1990, why this seminal paper is not cited?) with PHA-L tracing topographic projection from various areas in the CN to various areas in the IO. In this study, they proposed that the topographic relationship of the GABAergic nucleoolivary projection covers the entire cerebellar nuclei and the entire inferior olive nucleus, except for some exceptional parts such as the Dorsal cap of Kooy of the inferior olive. This idea seems to remain as a "Closed circuit" in Figure 6. However, the GABAergic projection from the super colliculus (SC) to the cdMAO belongs to the "Open circuit" in Figure 6. One of the problems of this manuscript is whether the cdMAO has the closed circuit type GABAergic projection from the MN is not examined or even mentioned besides the newly discovered GABAergic projection from the SC.

We thank the Reviewer for bringing this up, and we fully agree with most of this. However, we did mention (line 70 after resubmission) that the projection from MCN to cdMAO is predominantly excitatory, while referring to a recent paper of our lab in Nature Neuroscience, in which we extensively describe that this MCN-cdMAO projection is in fact not GABAergic, but excitatory. We now highlight this better in the main text to avoid confusion. In addition, we now also cite the excellent Ruigrok and Voogd (1990) paper in the main text. Please note that our Table only contains citations that directly address the inhibitory or excitatory nature of the projections to the IO (so the Table does not refer to this ref). Last but not least, please also note that both we and Wang and colleagues (2023; Nature Neuroscience) also tested to what extent the cdMAO also receives a closed-circuit type GABAergic projection from the MN. This is not the case, as the direct projection is excitatory and the indirect GABAergic projection from the SC innervates the side of the IO that projects to the other side; this is now also better highlighted in Figure 6 and the Main text.

Indeed, either case R127 or R128 of Ruigrok and Voogd, 1990, in which PHA-L is injected into the caudal and dorsal MCN, seems to be labeled axonal terminals in the cdMAO. These projections should be mentioned and compared with the data of the present study. Ruigrok and Voogd, 1990, also describe that the density of PHA-L-labelled terminals within the caudal MAO, group "beta", VLO and DMCC, and dorsal fold of the DAO was always lower compared to the heavy, massed projections observed within the rostral MAO, PO, DAO, and DM. This description may be noted in the present study.

Therefore, it should be studied (or explained) whether the cdMAO has the closed circuit GABAergic projection from a certain area of the contralateral MAO. If it does not have such a connection, then the meaning of such inconsistent organization of the nucleoolivary projection should be discussed.

As also highlighted above, we agree with the Reviewer that the excellent paper by Ruigrok and Voogd (1990) should play a more central role regarding this subject. To stress the importance of this work, we now discuss their findings in the second paragraph of the Discussion and explain how they fit well with our current findings as well as those presented in the recent paper from our lab (Wang et al., 2023; Nature Neuroscience).

Concerning the brain stem input to the caudomedial MAO, the projection from the SC to the MAO shown by Akaike (1986) matches or overlaps the projection by the present study. He seems to think that this projection is excitatory because the SC stimulation evokes short-latency climbing fiber firing in the medial crus II (Akaike, 1986). The present manuscript also shows non-GABAergic projection from the SC to the cdMAO (Fig. 4A, white asterisk). Which are predominant, excitatory, or inhibitory projections from the SC to the cdMAO? How do the authors explain the differences between Akaike (1986) and the present findings? Would there be other major excitatory projections to the cdMAO besides that from the SC?

We thank the Reviewer for bringing up this point. Unfortunately, given the anatomical dataset of the present study, we cannot make a direct comparison between the physiological strength of the GABAergic and that of the glutamatergic projection from the SC to the IO. However, it should be noted that stimulation of a glutamatergic input to an IO cell will most likely lead to an olivary action potential, whereas that of a GABAergic input is less likely to do so (Loyola et al., 2023; eLife). Moreover, stimulating simultaneously the glutamatergic and GABAergic input to an olivary cell, still elicits an action potential (Loyola et al., 2023); this takes probably place due to the asynchronous release of GABA in the IO, which delays this inhibitory input by about 50 ms (See Best and Regehr studies). Thus, observing short-latency CF firing after SC stimulation (as done by Akaike) does not mean that there is no GABAergic input. We now discuss these details in the Discussion, while still also confirming the presence of a strong glutamatergic projection from SC to IO, separate from the inhibitory MCN-SC-IO projection, in line with the physiological findings from Akaike.

(2) The cdMAO is the essential subarea of the IO in this study.

The definition of the cdMAO should be clearly described together with the spatial extent of the cdMAO.

In Figures, the presence of the GABAergic terminals in the cdMAO is shown only in a few coronal sections. The entire extent (rostrocaudal extent) of the cdMAO should also be shown (in the horizontal trajectory of the IO, for example, c.f. Figure 9 of De Zeeuw et al., 1996, Morphological correlates of bilateral....., the MAO part of Fig. 2 of Ruigrok and Voogd, 1990; Figure 14 of Akaike, 1986 and other papers).

It is also necessary to identify the cdMAO by indicating its cortical and nuclear target areas. Fujita et al. (2020) have indicated that the cdMAO project to the zebrin-positive areas in vermal lobule VII and medial paravermis in simple lobule, crus I, crus II, and paramedian lobule and the caudal dorsal part of the MCN by referring to some preceding studies. Do the authors agree with this?

This comment is highly appreciated. We now include the explicit statement that we indeed consider cdMAO to be the part of the olive as described by Fujita and colleagues (2020). Following careful comparison of the anatomical characteristics, we are confident in our identification of cdMAO versus that of ccMAO. In fact, the distinction is aided by the clear separation of the GABAergic SC-IO projection (to cdMAO) versus that of the glutamatergic projection, which also includes the ccMAO. Please note that there is a visible line in the raw data between the cdMAO and ccMAO when quantifying the glutamatergic and the GABAergic SC-IO synapses. While presenting the complete rostro-caudal extent of cdMAO in Figure 6 we now also indicate in the text that the figure panels encompass Bregma -7.76 until -7.56 according to Paxinos.

(3) Figure 2F-H is missing.

We apologize for the misnaming of the figures in the Results section. We corrected this mistake (it now reads "Fig. 1F-H").

(4) Lines 94-96: "We then confirmed in a transsynaptic tracing experiment that the MCN and lateral CN (LCN) both provide contralateral excitatory input to these GABAergic cells in the SC." On a related note, the projection from the cerebellar nuclei to the superior colliculus is shown as excitatory in Figures 3F and 6A. However, I could not find any data in this manuscript to support these descriptions.

We thank the Reviewer for this question. The reason for this interpretation is based on three sets of findings. First, Judd and colleagues (2021, eLife) did a brain-wide study on the GABAergic projections of the CN, but they were not able to find a GABAergic projection from the CN to SC; second, all long-range projections from the CN to the brainstem, other than the one projecting to the IO, have been shown to be excitatory (e.g., De Zeeuw and Ruigrok, 1994 Brain Res; Verveer et al., 1997 Brain Res; for Review see Novello et al., 2022 Cerebellum); and third, physiological studies have also shown predominantly an excitatory connection (see e.g., Westby et al., 1993; European J. Neuroscience). These arguments are now better explained in the main text, while citing the papers indicated above.

(5) Lines 248-249: Details of "our anterograde tracing experiment" should be explained, as there is no explanation in the Methods and Legend.

We thank the Reviewer for pointing out this omission. We now included these details in the Methods section as well as the Legends.

Minor Comments

Line 75: "all olivary dendrites of all olivary neurons" sounds redundant.

We agree with the reviewer and improved this sentence, by taking out the first "olivary".

Line 189, define "MilliQ".

We now included details on theMillipore machine.

Lines 129-130 and 140: The virus "pAAV-CAG-FLEX-rc" should be written in full without truncation. Its serotype should also be given.

We now added these details in the new version of the manuscript.

Line 132: The abbreviation "CTB" should be spelled out the first time it appears. The marker conjugated to CTB should be specified.

We now also added these details.

Line 253-254, "... input to the cdMAO from the hindbrain". Isn't this phrase awkward because the superior colliculus is in the midbrain?

We thank the Reviewer for pointing this out. We corrected this, while still highlighting that the SC is located posterior. We now conclude that all sources of the GABAergic projections to the IO are located in the posterior part of the midbrain or hindbrain.

Figure 1 and other figures: Insert space between digits and "micrometer", "200um" should read "200 um".

We corrected this.

Figure 1B, Why is the bilateral dorsal cap nuclei of the inferior olive (dorsomedial side) labeled in magenta?

The labeling is bilateral, because the injections traversed the midline in between the two dorsal cap nuclei. Please also note that the projection from the nucleus prepositus hypoglossi to the dorsal cap is bilateral (De Zeeuw et al., 1993 JCN). In our case too, we strongly labelled the nucleus prepositus hypoglossi bilaterally and their concurrent anterograde projections to the dorsal cap nuclei.

Figure 1D, E: These 3D models are very difficult to understand. Can they be revised somehow?

We now added directionality arrows and a zoom-out of the whole brain to aid interpretation of these images.

Figure 1H, How subareas d, c, b, and a were defined in the caudal MAO?

As now better explained in the main text, we follow the description by Fujita and colleagues (2020).

Figure 2: For better understanding, schematic panels (like Figures 1A, 1F and 3A) should be added for each type of experiment.

We now added the schematic panels for all experiments (2C, 4A).

Figure 2C, "fr" does not seem to be defined. Also, details of "retrograde Cre-expressing virus" should be provided.

Details of the virus have now been added in the Methods section.

Lines 279-281. The author says the LCN project to the SC. As far as I know, the ventral PIN also projects to the SC (Y Kurimoto, S Kawaguchi, M Murata, 1995, Cerebellotectal projection in the rat: anterograde and retrograde WGA-HRP study of individual cerebellar nuclei, Neurosci Res 22(1):57-71. doi: 10.1016/0168-0102(95)00874-s.; G W Westby, C Collinson, P Dean, 1993, Excitatory drive from deep cerebellar neurons to the superior colliculus in the rat: an electrophysiological mapping study Eur J Neurosci 5(10):1378-88. doi: 10.1111/j.1460-9568.1993.tb00924.x.; and other papers). The authors should also look at the ventral PIN as well as the LCN.

We thank the Reviewer for pointing this out. To accurately answer this point, we injected CTB in the lateral or medial SC and quantified the retrogradely labelled cells in all cerebellar nuclei. Indeed, we confirmed the targeted labeling in MCN and LCN, and also found some labeling in the PIN. This is now mentioned briefly in the Main text. However, we prefer to maintain our focus on the MCN as our primary investigation, and use the LCN labeling as control, since the LCN connections were always clearly segregated from those in the MCN, allowing for a rigid interpretation. The main scope of the current paper is on the MCN-SC-IO projection, as the laterality and polarity of this projection is unique and answers the questions that were left from the classical work of Mugnaini as well as from our current paper in Nature Neuroscience (Wang et al., 2023). This is now better highlighted in the Introduction.

Figure 3Bi, ii: Some cortical areas are much more densely labeled than the MCN or LCN. Does not this interfere with the authors' interpretation?

We thank the Reviewer for bringing up this point. In our injections we aimed to avoid any contamination of tracer injections on the ventral side of the CN, as the vestibular complex also provides GABAergic projections to the IO. As a consequence, the cerebellar cortex was also labeled in some of our experiments targeting the CN. However, primary transfection of the cerebellar cortex with an anterograde transsynaptic tracer that only jumps 1 synapse cannot result in aspecific expression of cre in the SC. We therefore feel rather confident that we are making the right interpretation. Still, to comply with this point, we explain our rationale on this point now better in the Results section.

Figure 4. The authors say A and B are symmetric and C is asymmetric. However, I cannot judge as they say. I do not think in these panels, in which the presynaptic component is filled with black reaction product,

To avoid ambiguous statements, we removed the terms "(a)symmetric" from the figure and legend.

Figure 4. The calibration bar is missing.

We added the calibration bar.

Line 321-322. "the postembedding GABA-labelling ... is significantly more than..." The meaning is not clear. "more" means "denser"? However, it seems to me that

We agree with the Reviewer that this statement is unnecessary and potentially confusing. We removed it from the figure Legend.

Figure 5, "DAO" may be more specified to either "ventral fold" or "dorsal fold", which would match with projection differences.

We agree with the Reviewer that this is an important differentiation. In the new version of Figure 5 we now explicitly indicate the dorsal and ventral fold of the DAO.

Line 381. "involved control" should read "involved in control" (?)

We improved this sentence.

Line 382: The phrase "a push-pull fashion" is vague. Perhaps more precise terms such as "synergistic" or "coordination of antagonistic effects" should be used. The same phrase in the abstract should also be reconsidered

We thank the Reviewer for this suggestion and incorporated this in the Main text. Yet, we prefer to keep the word "push-pull" in the abstract for reasons of conciseness and accessibility for layman.
